# Proceedings of the Seventh Annual Deep Brain Stimulation Think Tank: Advances in Neurophysiology, Adaptive DBS, Virtual Reality, Neuroethics and Technology

**DOI:** 10.3389/fnhum.2020.00054

**Published:** 2020-03-27

**Authors:** Adolfo Ramirez-Zamora, James Giordano, Aysegul Gunduz, Jose Alcantara, Jackson N. Cagle, Stephanie Cernera, Parker Difuntorum, Robert S. Eisinger, Julieth Gomez, Sarah Long, Brandon Parks, Joshua K. Wong, Shannon Chiu, Bhavana Patel, Warren M. Grill, Harrison C. Walker, Simon J. Little, Ro’ee Gilron, Gerd Tinkhauser, Wesley Thevathasan, Nicholas C. Sinclair, Andres M. Lozano, Thomas Foltynie, Alfonso Fasano, Sameer A. Sheth, Katherine Scangos, Terence D. Sanger, Jonathan Miller, Audrey C. Brumback, Priya Rajasethupathy, Cameron McIntyre, Leslie Schlachter, Nanthia Suthana, Cynthia Kubu, Lauren R. Sankary, Karen Herrera-Ferrá, Steven Goetz, Binith Cheeran, G. Karl Steinke, Christopher Hess, Leonardo Almeida, Wissam Deeb, Kelly D. Foote, Michael S. Okun

**Affiliations:** ^1^Department of Neurology, Norman Fixel Institute for Neurological Diseases, Program for Movement Disorders and Neurorestoration, University of Florida, Gainesville, FL, United States; ^2^Departments of Neurology and Biochemistry, and Neuroethics Studies Program—Pellegrino Center for Clinical Bioethics, Georgetown University Medical Center, Washington, DC, United States; ^3^J. Crayton Pruitt Family Department of Biomedical Engineering, Center for Movement Disorders and Neurorestoration, University of Florida, Gainesville, FL, United States; ^4^Department of Neurosurgery, Center for Movement Disorders and Neurorestoration, University of Florida, Gainesville, FL, United States; ^5^Department of Biomedical Engineering, Duke University, Durham, NC, United States; ^6^Department of Neurology, University of Alabama at Birmingham, Birmingham, AL, United States; ^7^Department of Biomedical Engineering, University of Alabama at Birmingham, Birmingham, AL, United States; ^8^Department of Neurology, University of California, San Francisco, San Francisco, CA, United States; ^9^Graduate Program in Neuroscience, Department of Neurological Surgery, Kavli Institute for Fundamental Neuroscience, University of California, San Francisco, San Francisco, CA, United States; ^10^Department of Neurology, Bern University Hospital and the University of Bern, Bern, Switzerland; ^11^Medical Research Council Brain Network Dynamics Unit, University of Oxford, Oxford, United Kingdom; ^12^Department of Neurology, The Royal Melbourne and Austin Hospitals, University of Melbourne, Melbourne, VIC, Australia; ^13^Medical Bionics Department, University of Melbourne, East Melbourne, VIC, Australia; ^14^Bionics Institute, East Melbourne, VIC, Australia; ^15^Department of Surgery, Division of Neurosurgery, University of Toronto, Toronto, ON, Canada; ^16^Institute of Neurology, University College London, London, United Kingdom; ^17^Edmond J. Safra Program in Parkinson’s Disease, Morton and Gloria Shulman Movement Disorders Clinic, Toronto Western Hospital, UHN, Toronto, ON, Canada; ^18^Division of Neurology, University of Toronto, Krembil Brain Institute, Center for Advancing Neurotechnological Innovation to Application (CRANIA), Toronto, ON, Canada; ^19^Department of Neurological Surgery, Baylor College of Medicine, Houston, TX, United States; ^20^Department of Psychiatry, University of California, San Francisco, San Francisco, CA, United States; ^21^Department of Biomedical Engineering, Neurology, Biokinesiology, University of Southern California, Los Angeles, CA, United States; ^22^Case Western Reserve University School of Medicine, University Hospitals Cleveland Medical Center, Cleveland, OH, United States; ^23^Departments of Neurology and Pediatrics at Dell Medical School and the Center for Learning and Memory, University of Texas at Austin, Austin, TX, United States; ^24^Laboratory for Neural Dynamics and Cognition, Rockefeller University, New York, NY, United States; ^25^Department of Bioengineering, Stanford University, Stanford, CA, United States; ^26^Department of Biomedical Engineering, Case Western Reserve University, Cleveland, OH, United States; ^27^Department of Neurosurgery, Mount Sinai Health System, New York, NY, United States; ^28^Department of Psychiatry and Biobehavioral Sciences, David Geffen School of Medicine at UCLA, Los Angeles, CA, United States; ^29^Department of Neurology, Cleveland Clinic, Cleveland, OH, United States; ^30^Center for Bioethics, Cleveland Clinic, Cleveland, OH, United States; ^31^Asociación Mexicana de Neuroética, Mexico City, Mexico; ^32^Medtronic Neuromodulation, Minneapolis, MN, United States; ^33^Neuromodulation Division, Abbott, Plano, TX, United States; ^34^Boston Scientific Neuromodulation, Valencia, CA, United States; ^35^Department of Neurosurgery, Norman Fixel Institute for Neurological Diseases, Center for Movement Disorders and Neurorestoration, University of Florida, Gainesville, FL, United States

**Keywords:** deep brain stimulation, stereoelectroencephalography, depression, Parkinson’s disease, tremor, optogenetics, local field potentials, neuroethics

## Abstract

The Seventh Annual Deep Brain Stimulation (DBS) Think Tank held on September 8th of 2019 addressed the most current: (1) use and utility of complex neurophysiological signals for development of adaptive neurostimulation to improve clinical outcomes; (2) Advancements in recent neuromodulation techniques to treat neuropsychiatric disorders; (3) New developments in optogenetics and DBS; (4) The use of augmented Virtual reality (VR) and neuromodulation; (5) commercially available technologies; and (6) ethical issues arising in and from research and use of DBS. These advances serve as both “markers of progress” and challenges and opportunities for ongoing address, engagement, and deliberation as we move to improve the functional capabilities and translational value of DBS. It is in this light that these proceedings are presented to inform the field and initiate ongoing discourse. As consistent with the intent, and spirit of this, and prior DBS Think Tanks, the overarching goal is to continue to develop multidisciplinary collaborations to rapidly advance the field and ultimately improve patient outcomes.

## Introduction

Since 2012, the annual Deep Brain Stimulation (DBS) think tanks have convened subject matter experts in neuromodulation research and clinical practice to exchange ideas, discuss developing technologies, address and plan for current and future challenges and opportunities in the field (Gunduz et al., 2015; Deeb et al., 2016; Rossi et al., 2016; Ramirez-Zamora et al., 2018, 2019). The Seventh Annual DBS Think Tank took place on September 8, 2019 (a virtual meeting held *via* Zoom Video Communications inc due to travel concerns and impediments caused by Hurricane Dorian). The meeting focused on advances in: (1) commercially available technologies; (2) the use of advanced technologies to improve clinical outcomes; (3) research in neuromodulatory approaches to treating neuropsychiatric disorders; (4) the use and utility of complex neurophysiological signals for advancing delivery of neurostimulation; and (5) ethical issues arising in and from research and use of DBS.

## Applying Advanced Neurophysiological Signals to Advance DBS Treatment

### Adaptive Deep Brain Stimulation in Parkinson’s Disease

Adaptive deep brain stimulation (aDBS) for movement disorders has been demonstrated to be effective during in-clinic, short term testing. Early studies suggested that beta activity may be a reasonable biomarker for PD clinical state (Kühn et al., 2006; Little et al., 2012a,b). aDBS uses unique neurophysiological signals to direct the delivery of stimulation to control motor symptoms (Supplementary Figure S1). The first human trials of aDBS used a subcortical beta signal and a fixed threshold with short time scales (Little et al., 2013, 2016a,b). This protocol targeted prolonged beta bursts and, through stimulation, shortened their duration (Tinkhauser et al., 2017a,b). The initial studies found that aDBS was more effective than conventional (cDBS) using blinded Unified Parkinson’s Disease Rating Scale (UPDRS) ratings (both unilaterally and bilaterally). Moreover, aDBS reduced speech side effects (acutely) and was appropriately responsive to levodopa medication (Little et al., 2016b). This approach has subsequently been shown to possibly prevent dyskinesia (Rosa et al., 2015; Arlotti et al., 2018).

The initial limitations of early studies were the post-operative microlesion effect and the brevity of stimulation. One post-operative study did however successfully stimulate for 8 h across medication cycles (Arlotti et al., 2018). Other studies have attempted to implement aDBS in the chronic phase, at battery change or in chronically implanted systems, and have shown aDBS to be as effective as cDBS, despite significantly reduced current delivery (Piña-Fuentes et al., 2017, 2019a; Velisar et al., 2019). Additional signals are being investigated, and dyskinesia has been associated with a narrowband gamma oscillation in the motor cortex between 60 and 90 Hz, with a similar but weaker oscillation in the subthalamic nucleus (STN) and strong phase coherence between the two (Swann et al., 2016). Successful control of hyperkinetic movements using an adaptive DBS design has been conducted with encouraging results (Swann et al., 2018b).

Notable was the use of a dual-threshold system and kinematic evaluation in a chronic fully implanted beta aDBS system. This approach was shown to be feasible and of practical utility in clinical settings (Velisar et al., 2019; Supplementary Figure S2). Direct kinematic assessments may be important for advancing aDBS and for preventing deleterious effects (although it remains to be determined if and to what extent small kinematic differences will impact clinical outcomes; Johnson et al., 2016). To date, there have been eight trials of aDBS in PD, and all trials have shown aDBS to be at least equivalent to cDBS in achieving relevant clinical outcomes. Ascertainment of whether this approach will be equivalently more effective in out-of-clinic (i.e., real-world) environments will require larger trials using fully implanted devices with embedded sensing capabilities. The use of aDBS for treating other conditions, including tremor and dystonia, has only begun to be tested, and initial findings suggest that this approach could be promising for treating these conditions. As well, the use of patterned stimulation using the neural activity phase rather than just local field potentials (LFP) amplitude is being considered (Cagnan et al., 2014, 2017; Piña-Fuentes et al., 2019b). It is unclear if aDBS would provide a clear advantage in reducing the burden and complexities of prolonged programming visits as additional time might be required to set up appropriate stimulation. This is an important but as yet unanswered question that remains the focus of future research. Overall, these studies provide a building body of support for the use and value of aDBS in treating a number of movement disorders.

### Sensing and Adaptive Loop Stimulation Using the Summit RC+S Platform: Early Experience

Chronically implantable neuromodulating devices that both sense brain activity and deliver stimulation (i.e., “bidirectional” interfaces) have generated excitement in the neurosurgical and neurology communities. Potential uses of bidirectional interfaces include the identification of electrophysiologic signatures of specific signs or symptoms of brain disorders, and the development of aDBS (Modolo et al., 2012; Swann et al., 2018b). However, the first-generation devices (that have been available since 2013) had limitations in signal quality, management of stimulation artifact, and the capability for continuous collection of brain data in home environments (Swann et al., 2018a). Toward closing these gaps, we have gained experience with a newly available second-generation device, Summit RC+S (Medtronic) in five patients with PD (Stanslaski et al., 2018). In our recent protocol, the Summit RC+S DBS was attached to a quadripolar depth lead (STN or Globus Pallidus Interna (GPi); for both stimulation and sensing), and a quadripolar paddle lead in the subdural space over the primary motor cortex (for sensing only). While this system was more customizable than previously used devices, the relative complexity of RC+S system necessitated a team approach to include software engineers to customize programs using the device application-programming interface (API). Summit RC+S provided substantial improvements over first-generation devices with respect to signal to noise characteristics, long term continuous data streaming, and interference from stimulation or other artifacts. These features permitted hundreds of hours of recording of electrophysiologic data that could be obtained in the home environment. Additionally, RC+S-obtained data can be synchronized offline to pair with external wearable sensors (such as smartwatches) to further characterize physiologic states. Our initial results showed canonical levodopa-related changes in subthalamic and cortical field potentials that have previously been demonstrated only using externalized leads in brief in-clinic recording periods. The use of LFP electrophysiologic signatures and classifiers made it possible to distinguish clinical states with high accuracy in out-of-clinic (i.e., home) environments (Supplementary Figure S3). We view this as a critical step in advancing aDBS toward a more useful therapy. Moreover, the capability for invasive neural recording over long periods of time in naturalistic environments will afford a novel method for acquiring basic neuroscientific data that can be used to guide subsequent bio-engineering and clinically relevant research directions and developments.

### Combining Directional DBS and Physiology Toward an Adaptive Loop Approach

aDBS and directional DBS (dDBS) are two recent technological innovations that have fostered new strategies to refine the DBS programming process (Modolo et al., 2012; Kühn and Volkmann, 2017). aDBS is a technique in which DBS output incorporates real-time sensing data *via* a feedback mechanism in order to guide stimulation delivery. dDBS refers to DBS leads with segmented electrodes that enable the generation of a spatially selective electric field directed toward a brain region of interest. These methods contrast with cDBS, which utilizes a ring-shaped electrode that can only generate electric fields symmetric about the long axis of the lead. Both aDBS and dDBS have been important to developing DBS optimization strategies that focus both on temporal domains (utilizing aDBS), and spatial domains (using dDBS). Studies of dDBS have reported that although DBS programming time can significantly increase with this new approach, there is also potential expansion of the therapeutic window (Contarino et al., 2014; Pollo et al., 2014; Steigerwald et al., 2016; Dembek et al., 2017; Rebelo et al., 2018; Ten Brinke et al., 2018).

LFP recordings from basal ganglia in PD patients using dDBS technology have indicated that beta signal strength is not homogenously distributed around segmented leads (Fernández-García et al., 2017; Tinkhauser et al., 2018). Selective stimulation of these regions with high beta power was associated with greater therapeutic benefit (Tinkhauser et al., 2018) and could serve as a physiology-based tool to optimize DBS contact selection. As technology continues to improve, combining dDBS with aDBS may afford improved clinical outcomes due to a wider therapeutic window, a more flexible selection of stimulation and recording contacts, and improved DBS programming time through the utilization of automated aDBS techniques.

### DBS Local Evoked Potentials: The ERNA

STN DBS for treatment of PD requires precise lead placement but marked variability between lead location among patients might cause difficulties optimizing stimulation parameters. The burden of programming has been increasing with the advent of current steering electrodes, and this technology has also increased the available parameter space. The use of a neuronal biomarker to guide electrode implantation could be beneficial for performing surgery, particularly if the patient is asleep. Such a biomarker could guide stimulation in at least two domains: parameter settings and adaptive control. The biomarker should localize to the STN, reflect the clinical state of the patient, modulate with DBS, possess reasonably fast correlation, and be reliably detectable.

Currently, beta oscillations are a commonly used biomarker metric (Wingeier et al., 2006; Little et al., 2013; Tinkhauser et al., 2018). However, these oscillations are small (<15 μV), and can be difficult to detect in and across certain conditions. In this light, we propose a new biomarker, evoked resonant neural activity (ERNA), which is significantly larger than the beta band (>100 μV) and can be reliably recorded across all conditions (Sinclair et al., 2018, 2019). ERNA is evoked and recorded from within the STN itself and is generated by a train of square biphasic pulses. After cessation of the pulses, the evoked response persists with a decaying oscillation. Both the amplitude and frequency seem to be components of the biomarker. Unlike beta oscillations, ERNA is an active signal created by stimulation, and cannot be recorded from resting-state activity. ERNA localizes to the STN (Supplementary Figure S4A) and is present under general anesthesia (Supplementary Figure S4D), suggesting a role to guide electrode implantation with patients asleep without intraoperative imaging (Sinclair et al., 2018). ERNA amplitude varies even within the STN, being maximal in the dorsal subregion where DBS is reported to achieve the greatest benefit (Horn et al., 2017; Sinclair et al., 2018; Supplementary Figure S4B) and electrodes that record larger ERNA amplitudes have been found to produce greater clinical benefit (Supplementary Figure S4C). These findings raise the possibility that the ERNA amplitude could help guide electrode contact selection. Moreover, the frequency of ERNA modulates during DBS (Supplementary Figure S4E), reducing on average from around 310 Hz pre-therapy to around 260 Hz when therapy reaches clinically effective levels (Sinclair et al., 2019). It is intriguing that the latter value is around twice the commonly employed applied STN DBS frequency of 130 Hz, though it is crucial to note that the plateau frequency that ERNA reaches with therapeutic DBS differs between individuals. Future work will assess whether the plateau frequency that ERNA reaches with therapeutic DBS could inform on the ideal DBS frequency to apply in individuals. During DBS, modulation of ERNA frequency correlates with the amplitude of beta oscillations (a useful surrogate of the clinical state of the patient; Supplementary Figure S4F; Sinclair et al., 2019). During programming, the ERNA could facilitate optimization of more precise settings, especially given that there is a larger amplitude in the dorsal motor region of the STN (Sinclair et al., 2018). This raises the possibility that ERNA frequency could be investigated as a biomarker to trigger adaptive STN DBS in PD.

### Multimodal Evoked Potential Elicited by DBS: New Candidate Biomarkers to Guide Novel Therapies

Optimal DBS therapy is challenged by complex new technologies and heterogeneous diseases, leading to two important questions. Which biomarkers are the most suitable, and where should one record? Multimodal recordings of stimulus-evoked activity elicited by DBS paired-pulse studies leverage electroencephalography (EEG), electrocorticography (ECoG), and DBS LFP data. Data from these recordings can be used to help answer these questions. Our group recorded EEG potentials from STN (i.e., in PD) and thalamic (i.e., in essential tremor) neurons during therapeutic stimulation. Stimulation generated a polyphasic event-related potential in the ipsilateral sensorimotor cortex, with peaks at discrete latencies beginning less than 1 ms after stimulus onset (Walker et al., 2012a,b). There are marked differences in evoked potential (EP) morphology between effective and ineffective stimulation, with high-frequency activity occurring after ineffective stimulation. Avoiding capsular side effects (elicited by stimulating the surrounding internal capsule) can also be important when recording.

We also evaluated ECoG responses recorded from the primary motor cortex, following paired-pulse stimulation. As the interstimulus interval between the conditioning stimulus (CS) and the testing stimulus (TS) diminished, the latency increased and amplitude decreased, and the evoked response disappeared entirely as the interval fell below 1 ms. These effects highlight the relative and absolute refractory periods of neurons. DBS LFP recordings stimulate from the two inferior contacts and record from the two superior contacts of the depth lead. They show a response with a latency of 350 μs, as well as ERNA (Sinclair et al., 2018). As the CS and TS approach become more proximate, there is evidence of relative and absolute refractory periods as the latency increases and amplitude decreases, with the evoked response and ERNA disappearing as the two stimuli converge. With trains of stimulation, the ERNA shows remarkable facilitation at 500 Hz and at the clinically relevant frequencies of 100–250 Hz.

When co-recorded, EEG, ECoG, and DBS LFPs show latencies of 1 ms, 1 ms, and 350 μs, respectively, with each modality showing an increasing amplitude of the response by an order of magnitude when measured from scalp to subcortical recordings. All modalities show relative and absolute refractory periods consistent with the activation of neural structures. These responses were consistently observed across all modalities. In a recent report, our group presented evidence that DBS-evoked responses, including those at extremely short latencies, have a neural origin beneath or very near the ECoG strip over the ipsilateral premotor and motor cortex (Awad et al., 2020). It is proposed that the most likely underlying mechanism responsible is non-synaptic, retrograde activation of cortical neurons whose axons project to the subcortical stimulation site. Other factors might impact the spatiotemporal patterns of cortical activation by DBS and additional computation and basic science experiments should focus on a comprehensive, systems-level and physiological understanding of DBS. Relevant discussion at the time of the meeting questioned the use of this technique in clinical practice. At the present time, no clinical decisions are made by the University of Alabama team using this research protocol. Parameters are selected based on the most effective clinical response intraoperatively using a bipolar configuration with standard STN pulse width and frequencies.

### Neuronal Sources of Evoked Potentials

SNT DBS produces EPs both locally (i.e., in STN; sEP) and in the cortex (cEP). EPs can be regarded as a possible biomarker of neural responses to stimulation, but the origin of EP activity is not wholly clear. To address this issue, we used computational models to better understand the neural elements involved in—and contributing to—EPs.

The cEP included short-latency positive (P1), intermediate latency negative (N1), and long-latency positive (P2) responses in the rat (Kumaravelu et al., 2018) and human models (Walker et al., 2012a). Using a computational model of the thalamocortical (TC) network (Traub et al., 2005) to decipher the origin of cEPs (Kumaravelu et al., 2018), DBS was simulated by activating layer 5 pyramidal axons (antidromically) and applying inhibitory postsynaptic current to the thalamus (orthodromically), thereby mimicking hyper direct and indirect pathway activation, respectively. Model-based cEPs matched well with cEPs obtained from both rats and humans. P1 and N1 responses were due to the direct and recurrent activation of L5 pyramidal neurons, respectively, while P2 responses arose from polysynaptic activation of L2/3 pyramidal neurons following a cortico-thalamocortical loop. Antidromic activation alone can faithfully reproduce cEPs. Understanding anatomical pathways of cortical modulation can be used to optimize therapeutic targets, and cEPs may aid in electrode placement, selection of stimulation parameters, and/or in adaptive (closed)-loop control.

Unlike thalamic DBS EPps (Kent et al., 2015), sEPs were polyphasic and highly stereotyped (Grill et al., 2015; Sinclair et al., 2018). Implementing a 3-dimensional biophysical model of the STN-GPe subcircuit enabled the determination of the origin of sEPs. Model-generated sEPs for 45 Hz and 130 Hz DBS were similar to sEPs from humans with PD, indicating the involvement of STN-GPe interactions and hyper direct axons. The early positive phase resulted from antidromic STN excitation, while the early negative phase reflected strong inhibition by local pallidal terminals. The high-frequency oscillations occurring after the DBS pulse were caused by quasi-periodic pallidal inhibition. As with cEPs, sEPs reveal functional connectivity, and may also be useful as a guide for lead placement or as signals for adaptive-loop control.

## Advancements in Recent Techniques in Neuromodulation: Emerging Brain Targets, Use of Multiple Leads and Stereoelectroencephalography (SEEG)

### Emerging DBS Targets in Non-motor Disorders

As the number of refereed publications reflects, the field and use of DBS have increased from the late 1990s to the present, as evidenced by less than 100 published papers to over 1,000 papers published per year (Lee et al., 2019). To characterize this growth and to describe the state of the field, a comprehensive overview was presented through data obtained using ClinicalTrials.gov. At the time of this Think Tank, there were 422 registered DBS trials. In recent years, there have been about 40 new studies entered per year. The purpose of these studies has varied from large classical trials for new indications to investigations focused on more novel uses and protocols of DBS (e.g., mindfulness during DBS to improve patient comfort). The majority of studies have been observational or early phase studies, with less than 10% being interventional phase III-IV trials. Approximately 60% of studies addressed the use of DBS for movement disorders, with other areas of interest including psychiatric conditions (e.g., obsessive-compulsive disorder, depression, etc.), cognitive disorders (Alzheimer’s disease, non-Alzheimer’s dementia, etc.), pain (e.g., headache, neuropathic pain), epilepsy, and others (e.g., tinnitus, lower urinary tract symptoms, etc.).

At present, 28 cerebral targets are utilized in DBS according to this registry. These targets (inclusive of multiple sites being used to treat the same disorder) included: PD [nucleus basalis of Meynert, STN, GPi, ventral intermediate thalamic nucleus, pedunculopontine nucleus (PPN)], depression (subgenual cingulate gyrus, habenula, medial forebrain bundle, inferior thalamic peduncle, ventral capsule/ventral striatum, nucleus accumbens and anterior limb of the internal capsule;), and obsessive-compulsive disorder (medial thalamus, inferior thalamic peduncle, ventral capsule/ventral striatum, nucleus accumbens, anterior limb of the internal capsule, bed nucleus of the stria terminalis and the STN; Budman et al., 2018; Lee et al., 2019). Nearby targets for the same disorder often may be no more than a few millimeters apart. As the number of disorders and brain targets under investigation continues to expand, improved neurosurgical targeting accuracy and current steering will be required to better define these targets and to delineate nuclei vs. pathway stimulation. As neurosurgical targeting becomes increasingly precise (and “personalized”), direct comparisons using crossover study designs will better inform the field. Advances in research and technology in the field of DBS might allow not only treatment of new disorders but also improve our understanding of the pathophysiological mechanisms of neuropsychiatric conditions and exploration of novel DBS targets using invasive and non-invasive approaches.

### Emerging DBS Targets in Motor Disorders

PPN DBS continues to be investigated for its role in the freezing of gait in PD. Interest is driven largely by the PPN’s documented relationship to the mesencephalic locomotor region. Variability in PPN DBS outcomes has been attributed to electrode targeting, patient phenotype, outcome measure, and duration of benefit (Thevathasan et al., 2018). As regards electrode targeting, a recent study summarized PPN anatomy and targeting terminology and noted domains of uncertainty that require further investigation and elaboration (Hamani et al., 2016). Outcome measures have previously included the UPDRS as well as the use of questionnaires that specifically assess gait and freezing. While these are certainly valid, viable and of value, we opine that employing objective measures will likely facilitate the most useful and reproducible approaches. However, procedures used and data gained in gait laboratories may not represent real-world environments, and this variability of such environmental circumstances may be a critical factor for inducing freezing of gait. Indeed, many patients do not freeze (or display improvement of freezing) when in laboratory settings. Hence, reproducibility under laboratory conditions (i.e., efficacy) may not be a reliable measure—or predictor—of real-world effectiveness.

As well, it is important to note that the current state of knowledge on PPN DBS is derived from relatively small-scale studies, and there remains much room for continued (and broadened) investigation. For example, a single case of cyclical PPN DBS (DBS turned off overnight) provided extended benefit beyond that produced by non-cyclical PPN DBS (Stefani et al., 2013). Other approaches, including dual stimulation or dual-frequency stimulation of the STN and substantia nigra pars reticulata (SNr) for resistant axial motor impairment as well as spinal cord stimulation (Weiss et al., 2013; Samotus et al., 2018; Valldeoriola et al., 2019) and adaptive loop DBS has also been undertaken. Additional small uncontrolled studies are exploring other targets for refractory tremors including the caudal Zona incerta and the centromedian and parafascicular nuclei of the thalamus. Combined PPN and caudal zona incerta stimulation, stimulation of the Centromedian and Parafascicular nuclei of the thalamus, and extradural motor cortex stimulation are other targets currently being investigated for the management of refractory axial symptoms in PD (Anderson et al., 2017). While outcomes are promising, these (and additional) approaches will require further study.

### Using Multiple DBS Targets

Electrodes with multiple contacts may be used to simultaneously reach many targets oriented along a dorsal-ventral axis. Exemplary cases have included stimulation of the STN and thalamus for mixed PD and ET-like action tremor (Baumann et al., 2012), as well as the aforementioned STN and SNr stimulation for gait and balance. Different electrode designs, such as the Boston Scientific 8-contact device (spanning 1.5 cm) may provide greater flexibility for simultaneously targeting multiple sites for DBS. This device could enable the option to stimulate the SNr, STN, posterior subthalamic region, and thalamus with a single trajectory.

A small double-blind, crossover study is currently in progress to test stimulation at many of these sites. Additionally, it is known that both GPi and globus Pallidus externa (Gpe) DBS may be beneficial for patients with PD (Vitek et al., 2004). Therefore, it may be of value to synergistically target the GPi and GPe, and double stimulation may be more beneficial than stimulation directed to either target alone. Further, a trial is currently underway that employs simultaneous targeting of GPi and the nucleus basalis of Meynert to affect motor and cognitive symptoms in PD (NCT02589925). Novel electrode designs may also prompt further innovation in multiple targeting approaches. The trend in the field has been slowly moving toward the use of multiple DBS targets. These can now be accessed with a single DBS device, and we believe that such developments will afford greater possibility, accuracy, and effectiveness for targeting multiple signs, symptoms, and dimensions of a variety of neuropsychiatric (and other) disorders.

### A Randomized Controlled Trial of Personalized Adaptive-Loop DBS to Ameliorate Treatment-Resistant Depression

Major depression is a leading cause of disability worldwide. As well, not all patients respond to pharmacological standard of care interventions. Thus, we believe that those patients resistant to current standards of care might benefit from the use of novel neurotechnologies like DBS. However, randomized control trials of using continuous DBS of pre-selected brain locations to treat major depression, while relatively efficacious, did not yield statistically significant results (Kisely et al., 2018). Difficulties in treating depression with DBS may be related to the complex and heterogeneous nature of this disorder. A personalized aDBS approach that takes into account inter-individual differences could address these challenges. We have designed a 3-stage randomized controlled trial for intervention against treatment-resistant depression (i.e., the PRESIDIO trial) that will test the feasibility, safety and initial efficacy of personalized adaptive-loop DBS with the NeuroPace Responsive Neurostimulation (RNS) System (NCT04004169). Enrollment will include 12 adults with severe treatment-resistant depression who have been unresponsive to four trials of antidepressant medication and psychotherapy.

During the first stage of the study, subjects will undergo temporary implantation of electrodes and will be tested for biomarkers and conduct stimulation endpoints in order to guide target selection. In the second stage, the NeuroPace RNS System will be implanted with lead placement guided by results achieved during stage 1. Subsequently, the short-term and long-term efficacy of personalized adaptive-loop DBS treatment will be examined. The primary outcome measure will be a change in the Montgomery-Asberg Depression Scale (MADRS) score after 6 weeks of treatment stimulation compared with 6 weeks of sham stimulation. Safety will be monitored and recorded throughout the trial. This trial will provide a first-time opportunity to obtain direct recordings of neural networks involved in—and focal to the treatment of—treatment-resistant depression. This trial may enable the identification of quantitative markers of depression and afford an understanding of their dynamics.

### Stereoelectroencephalography for DBS Targeting in Pediatric Patients

The targets of DBS treatment of pediatric secondary dystonia vary across patients. The current standard of care to confirm DBS targets in secondary dystonia involves intra-operative microelectrode recordings, which entail waking the patient during surgery, and therefore necessitates anesthesia without intubation. However, there are children who are unable to tolerate this standard technique because of hyperkinetic dystonic movements and/or airway issues. To overcome this challenge, we have developed a novel 3-stage neuromodulation approach to determine clinically effective DBS targets (Sanger et al., 2018). At stage 1, patients are implanted with stereoelectroencephalography (SEEG) electrodes (Adtech mm16c) in ~10 potential targets for 5 days of testing and observation. This testing involves single-unit recordings, confirmation of locations with peripherally- and intracortical-EPs, identification of the therapeutic window (efficacy and side effects), and the effect(s) of stimulation on specific tasks. These data can then be used to determine whether to proceed with the second stage (i.e., implantation of DBS leads) and third stage (i.e., implantation of the pulse generator). Typically, approximately 50% of patients with secondary dystonia who undergo DBS show clinically relevant improvement. In comparison, 88% of subjects showed improvements on dystonia scales (Burke-Fahn-Marsden Dystonia Rating Scale, an average improvement of 10 points), and no subjects had worsening of signs and symptoms when utilizing this neuromodulation approach. Overall, this approach was well-tolerated, but there was a significant microlesion effect that seemed to resolve within a week, and this effect may limit the number of thalamic leads to six. Moving forward it will be important to focus on smaller electrodes that may induce smaller microlesions and a simplified conversion to permanent leads.

### Use of SEEG for Early Evaluation of Novel Targets and Indications

Classical DBS targets have primarily been based on decades of clinical experience using therapeutic lesions. Advancements in neuroimaging, recording electrodes and overall insight to the node and network function in the brain (both in health and disease) have prompted consideration and exploration of new targets for DBS to treat an expanding number of pathologies. However, new targets and indications lack the validation necessary to move from promising preclinical studies to rigorous clinical trials. One possible means to address and close this gap between preclinical studies and clinical trials is SEEG. As discussed above, SEEG involves the temporary (<3 weeks) implantation of multiple depth electrodes with an array of contacts in different deep brain regions. SEEG electrodes are similar in size and impedance to traditional DBS electrodes, can be implanted into many regions simultaneously, and can utilize externalized stimulators to test novel waveforms and adaptive-loop paradigms. This technique has been used in epileptic patients since the 1960s to record the onset and early spread of seizures (Youngerman et al., 2019). This history of being a well-tolerated and safe technique affords SEEG a particular advantage when considering its use in other protocols and paradigms. Still, it is important to note that the use of SEEG in epilepsy patients may not provide direct comparative value for the use of this technique with DBS.

Yet this too provides a window of opportunity to assess the efficacy, effectiveness and relative research and clinical value of SEEG in tandem with DBS. Toward such ends, the SEEG may be used: (1) for research in patients without epilepsy; (2) with implantation of other (additional) electrodes for studying effects in recording and stimulating brain regions of patients with other (non-epileptic) disorders; (3) for clinical purposes; and (4) to modify the trajectory of clinical electrodes. Evidently, there are ethical issues that must be considered when deciding upon an implantation intent and strategy (e.g., institutional review board (IRB) approval, informed consent, FDA exemption, and scientific rationale rigor). Some examples of recent use of SEEG with DBS include treatment of tinnitus; developing brain-computer interfacing of the primary motor cortex; DBS of the dorsal hippocampal commissure for treatment of mesial temporal lobe epilepsy; DBS of the hypothalamus for treating hypertension; theta burst stimulation/of the fornix region for treatment of post-traumatic memory loss; and DBS of the rostral cingulum bundle for treatment of bipolar disorder. Thus, with proper approval, SEEG can provide a powerful tool to evaluate short-term stimulation of novel DBS targets, and in such ways, may be instrumental to the discovery of new methods and applications of DBS.

## New Developments in Optogenetics and DBS

### Thalamocortical (TC) Physiology in Autism

To understand how different gene mutations lead to a common behavioral phenotype, it is necessary to gain insight into the ways that diverse genetic etiologies converge at the level of neuronal circuit physiology, and how changes in these circuits are involved in behavior. Previous studies have identified prefrontal circuits that are operative in symptoms and signs of autism spectrum disorder (ASD; Cheon et al., 2011; Kalmbach et al., 2015; Demetriou et al., 2018; Brumback et al., 2018; Maximo and Kana, 2019). The integrity of the reciprocal circuit, from the thalamus to the prefrontal cortex (PFC), is required for many prefrontal-dependent behaviors (Parnaudeau et al., 2018). However, the ability to target these reciprocal prefrontal-TC circuits for neuromodulation is hampered by the lack of understanding of thalamic cell types (Rikhye et al., 2018). Apropos of this paucity of understanding, our current work focuses on single-cell electrophysiology of specific mediodorsal (MD) thalamic neurons that provide ascending input to the PFC, with emphasis upon the ways that these neurons are affected in a model of the autism-associated Fragile-X syndrome (FXS). Using a mouse model, retrograde tracers were stereotactically injected into the medial PFC to fluorescently label neurons in the thalamus that project to PFC. Using acute brain slices, whole-cell patch-clamp recordings were taken from visually-identified TC neurons in MD. It was observed that MD→mPFC neurons divide into two populations based on the presence or absence of a prominent conductance mediated by hyperpolarization and cyclic nucleotide-gated (HCN) channels (“voltage sag”). It was hypothesized that these two populations (“High Sag” and “Low Sag”) may be globally affected in autism models, or alternatively, that one population would be selectively impacted. To test this, recordings from these two populations of MD→mPFC neurons were taken in acute brain slices from FXS mice and control littermates. It was observed that in FXS mice, High Sag neurons were hypoexcitable; whereas Low Sag neurons were relatively unaffected. This mirrors findings of abnormal excitability in High Sag mPFC→MD neurons as previously described by our group and others. Ongoing studies aimed at obtaining *in vivo* recordings of LFPs and optogenetic manipulations will evaluate how differences in High and Low Sag neuron physiology influence TC oscillations and behavior. It is hoped that localizing symptoms and signs to specific circuits will help to create circuit-level therapies regardless of the genetic cause of the disorder.

### Neural Circuit Mechanisms of Memory Retrieval: Toward Mechanistic Insights and Therapeutic Targets

A current approach to understanding memory involves activating populations of cerebral neurons in order to examine how specific circuits and networks interact with the hippocampus to form, store and retrieve information. Our studies are aimed at identifying frontal brain areas and networks that could contribute to top-down (i.e., cortico-hippocampal) vs. bottom-up (i.e., Hippocampo-cortical) memory processing. Such top-down networks are indicative of storage pathways that are relatively independent of acquisition pathways, and these pathways could prove to be viable targets for DBS-based therapy for Alzheimer’s disease and/or Post-Traumatic Stress Disorder.

This technique involves injecting a retrograde virus to the hippocampus to identify direct inputs, including a direct prefrontal- -hippocampal pathway. Using tracer technology, it was demonstrated that these inputs were monosynaptic, and electrophysiological patch recordings revealed a prevalence of direct short-latency excitatory transmissions (Rajasethupathy et al., 2015). Optical activation of the prefrontal inputs to the hippocampus suggested the existence of prefrontal cortical mechanisms that could drive goal-directed memory retrieval in the hippocampus. Further experiments using *in vivo* calcium imaging of the hippocampus, paired with optogenetic activation of prefrontal inputs elucidated that behavioral training fortified hub neurons in the hippocampus that exist within an otherwise uncorrelated neural ensemble. These hub neurons were preferentially targeted by top-down prefrontal inputs and appear to act as conduits to recruit other domains of the memory network. These results suggested that plasticity in the prefrontal cortico- hippocampal network may contribute to PFC engagement of (the most recently active) memory encoding cells to enable future rapid retrieval of important memories. These insights into PFC-hippocampal memory networks may be important to the development of next-generation neuromodulatory approaches to learning and memory acquisition, preservation, and retrieval.

### Optogenetic STN DBS

The mechanisms underlying the therapeutic effects of STN DBS for PD are still poorly understood. The anatomical heterogeneity of brain tissue is such that DBS can modulate the activity of multiple neuronal elements in the STN, as well as in surrounding regions. Optogenetic techniques that enable cell-type-specific activation allows assessment of the behavioral effects of selective stimulation of STN local neurons and hyper direct pathway axons (Gradinaru et al., 2009). Selective activation of STN excitatory neurons is not effective for treating Parkinsonian symptoms in the unilateral 6-OHDA lesioned rat model of PD (Gradinaru et al., 2009). However, this conclusion may have been influenced by the slow response kinetics of channelrhodopsin-2 (ChR2) precluding generation of the regular high rate activity required for symptom relief (McConnell et al., 2012, 2016), and it remained unclear whether STN local cells contributed to the therapeutic effects of DBS.

Therefore, we re-examined the role of STN local cells in mediating the symptom-relieving effects of STN DBS using a much faster opsin: Chronos (Klapoetke et al., 2014). Optogenetic stimulation of Chronos-expressing STN cells at 130 Hz reduced pathological circling behavior, in contrast to results obtained using the much slower ChR2 opsin. Furthermore, optogenetic DBS of STN with Chronos was strongly dependent upon stimulation rates: high-frequency DBS (75, 100, 130 pps) relieved ipsilateral turning; while low rates (5 and 20 pps) were ineffective. In addition, optogenetic STN DBS at 130 pps corrected the bias to use the unimpaired forepaw in forelimb stepping; while the low rate (20 pps) DBS was not effective. These results indicated that direct optogenetic stimulation of STN neurons was effective in treating the symptoms of parkinsonism in the 6-OHDA lesion rat, provided that a sufficiently fast opsin was used. These findings highlight that the kinetic properties of opsins can strongly influence the effects of optogenetic activation/inhibition, and therefore must be considered when employing optogenetics to study neural stimulation.

## Holographic Deep Brain Stimulation; The Combinatory Use of Mixed, Augmented, and Virtual Reality and DBS

Virtual reality (VR) and augmented reality (AR) are increasingly being utilized for research and clinical applications. Technologies such as the Oculus VR system, the Microsoft Hololens, and other VR and AR systems enable enhanced visualization of neuroanatomy within a 3-dimensional (3-D) environment. Currently, most neuroanatomy has been presented in 2-dimensional (2-D) images utilizing generic axonal pathways that have been derived from textbook illustrations. This approach has not been sufficiently accurate to understand the complexity of axonal pathways and the effect of stimulation as applicable to deep brain neuromodulatory approaches. The Microsoft Hololens was first demonstrated in 2013. The Hololens enabled researchers to render 3-D visualization and allowed multiple users to interact with a model while maintaining interaction with each other^1^.

The first step in developing an accurate 3-D model of an axonal pathway atlas for DBS is to reconstruct tractographically-based pathways in basal ganglia. Layers of cortical surface rendering and vasculature can be added to the holographic 3-D model to increase the interactivity within and between users (Petersen et al., 2019; Supplementary Figure S5). These components allow neurosurgeons to better understand the different pathways within, between, and across brain structures. After the structures and axonal pathways are constructed, users can adjust the electrode trajectory to display different DBS lead insertions. Combining the trajectory and volume of tissue activation facilitates the visualization of structures and pathways that potentially would be activated by stimulation. This method can be used to enhance patient-specific surgical planning. Holographic visualization provides a new medium for creating axonal pathway models that we believe will certainly advance the scientific understanding and clinical utility—and value—of DBS.

### Using VR for Patient Engagement

The recent technological advancement coupled with the increased presence of social media has afforded growing opportunities for engaging and interacting with neurosurgical patients. In some circumstances, we believe that it is important to initiate the engagement of patients prior to commencing clinical care. To maximize pre-clinical patient engagement, we have developed informative social media and/or online websites that use AR simulation to provide high fidelity examples of prior surgical cases (Steinberger et al., 2020). This process allows patients to view and acquire gain knowledge of surgical procedures prior to the consultation, which can fortify patients’ level of familiarity and comfort with the procedures to be implemented. Of course, it is important to obtain patient consent if and when sharing information on social media. As well, when sharing information using simulation for social media engagement, caution should be exercised to avoid any patient identifiers.

The surgical team should explain how the AR simulation is created and how the surgical plan will be based upon the simulation. Surgical Center, an FDA approved surgical planning software, is currently being used for all AR simulations at the Mt. Sinai School of Medicine. The software includes 3-D modeling of the brain, a patient-specific navigation system, and a non-distracting heads-up display. Using these technologies, surgeons can develop a tailored consultation based on types of surgery within the nervous system. AR simulation is increasing patient satisfaction and retention. For example, after using AR for patient consultation, patient satisfaction with (i.e., -Press Ganey Scores), and confidence in the surgical team was shown to increase. A pilot study at Stanford studied the effect of neurosurgical VR services on patient satisfaction and revealed that overall more positive evaluations of surgical experiences (Collins et al., 2018). Currently, a research study is underway to assess the effect(s) of AR simulation on several metrics of neurosurgical patient satisfaction due to AR. While contributory to an increased appreciation for, and relative value of the potential uses and benefit of AR-based neurosurgical simulations, there is also a need for large-scale randomized trials to further explore patient engagement, experience and satisfaction when using this technology.

### Using VR With Local Field Potential Acquisition

Beyond surgical planning and patient orientation, VR can be used to simulate real-world environments while simultaneously studying neural signals. One problem with current methods for measuring and/or modulating brain activity has been movement artifacts that may obscure the signal. In combination with wireless chronic recording and stimulation devices such as Neuropace RNS, the Medtronic Percept, and the Medtronic RC+S, VR has facilitated the development of simulated tasks that are naturalistic and therefore more ecologically valid in order to study the neural signals relevant to DBS therapies in awake behaving humans (Collins et al., 2018).

To accomplish this goal, Topalovic et al. (2019) have developed a wireless control and synchronization system for the Neuropace RNS system using a Raspberry Pi equipped with network synchronization. This is a lightweight portable system including the Neuropace programmer and accessory that can communicate with the neurostimulator. While working on the synchronization system, a challenge arose due to telemetry-induced artifacts in scalp EEG. Although the artifacts could be filtered from the signal, it was more beneficial to utilize a custom USB switch to turn off telemetry after the recordings start. This procedure prevented injecting artifacts into the neural recordings. Layered atop the synchronization system and VR was the latitude to add external biometrics using eye-tracking and inertia sensors to capture the comprehensive behavioral data simultaneously during neural recordings.

This system is not specific to the Neuropace RNS system, and it can be adjusted for use in another system by simply connecting the sensing program to a Raspberry Pi interface. This procedure allows for full integration. We have found this system to be capable of integrating VR and LFPs. Additionally, Aghajan et al recently published their work using VR/AR in combination with Neuropace RNS to study spatial memory (Aghajan et al., 2017). This platform enables novel methods to study intracranial EEG activities during freely moving tasks with naturalistic behavior under experimental control.

## Advances in Commercial Technology and Future Directions

### Towards Adaptive Therapies in DBS

In recent years, DBS technology has evolved to improve patients’ clinical outcomes and experience and interactions with the device. There are several mechanisms that can be employed to improve patient results, including calibration (optimizing parameters for specific patients and symptoms), lead localization, and the use of adaptive (closed-loop) technology. We opine that the improvement of aDBS applications will be particularly important—and necessary—to bridge this technology from research into clinical practice.

aDBS uses a marker to potentially trigger multiple stimulation parameters to improve the outcomes of stimulation. An example of this technology is provided by a Medtronic system, the Percept PC+S, that is capable of aDBS approaches for both research and clinical applications. The device offers state-of-the-art stimulation capabilities and includes a sensing engine for recording during stimulation. The device was designed to offer flexible configurations and real-time recording with longer telemetry distances. The device is smaller than other units and has longer battery life. Utilizing the PC+S real-time sensing and controlling capabilities allows aDBS applications. However, iterative evidence of efficacy will be required to clear this system for clinical use.

Previous approaches in aDBS focused on disease indications (e.g., PD, essential tremor, dystonia, epilepsy, Tourette syndrome, etc.,) For each indication, the initial approach has been to identify a signal of interest and to create control parameters (i.e., how the sensing signal triggers the stimulation). Controller classes based on amplitude, triggered response, desynchronization patterns, and other settings can be used group or categorize applications. The goal of centering use on controller classes is to develop broad evidence of safety and utility in order to expedite broader access to these technologies without the limitations incurred by simply using narrow disease-based applications or signals of interest.

### Directional DBS: Looking Back to Look Ahead

Previous DBS Think Tanks have identified key limitations with the technologies available at the time. Technologies under development were evaluated for their potential to serve unmet needs, and for each experimental approach, the most pertinent unknowns were identified. For example, the use of a segmented lead to provide “directional” or axially-asymmetric stimulation fields was one such development that was assessed in prior Think Tanks (Deeb et al., 2016; Ramirez-Zamora et al., 2018). In this regard, three important questions were posed: (1) would dDBS achieve a wider therapeutic window?; (2) how would stimulation through smaller, higher impedance electrodes affect power consumption?; and (3) how many segmented contacts would need to be activated, and would current fractionalization technology (e.g., multiple independent current controllers or temporal fractionalization through multi-stimulation sets/ interleaving) be required to achieve effective “steering” of electric fields?

The Abbott-sponsored study of directional vs. omnidirectional (conventional) stimulation (PROGRESS) provides the first high-quality evidence to address these clinically pertinent questions. This study prospectively enrolled 234 patients with STN DBS across 32 actively enrolling sites in Europe, the United States, and Australia. The primary outcome measure, therapeutic window, was evaluated in a randomized, double-blinded manner, 3 months after study enrollment. Secondary endpoints (patient and physician preference, on-meds UPRDS III) were evaluated in a sequential single-arm cross-over design: all patients received 3 months of omnidirectional stimulation followed by 3 months of directional stimulation. A performance target of 60% of patients with gain in therapeutic window on at least one side with dDBS was defined—in agreement with the European regulatory agency—as the superiority end-point. Primary endpoint data were available for 202 patients at the time of this year’s (2019) Think Tank. Of those evaluated, 90.6% of patients achieved a wider therapeutic window with dDBS (with a mean 40% gain in therapeutic window), thereby surpassing the superiority endpoint. Additionally, 86.6% of patients achieved a superior therapeutic window with the activation of a single segment compared to omnidirectional stimulation.

Directional stimulation achieved similar benefits at significantly lower therapeutic current strength (39% lower compared to conventional stimulation), which can have a meaningful positive impact on IPG lifespan. The programming approach prescribed as part of the protocol: prioritizing single segment activation and small step size for amplitude increments during a monopolar review, may importantly contribute to these results.

### Focusing on Improving DBS Outcomes

Boston Scientific has been developing technologies to improve DBS outcomes and accelerate programming. One of these products, developed with Great Lakes NeuroTechnologies, is Kinesia StimPoint: a tablet-based solution for augmented programming. This software presents a 2-D plot (stimulation location vs. amplitude) used to test, score, and view the clinical outcome of stimulation. Scores can be entered manually, or automatically using integrated accelerometer-based objective measures. This process updates the 2-D plot to reveal a stimulation response surface, and an algorithm suggests next settings. Boston Scientific is working to add support for directional leads to StimPoint. Programming may also be aided using patient imaging data paired with 3D stimulation models as in the Guide XT software, developed with BrainLab. When available, the combination of surgical, imaging, and stimulation response priors with real-time clinical response may further assist programmers.

Prior experimental and modeling studies have demonstrated that using hyperpolarizing or depolarizing pre-stimulation pulses affects stimulation outcome, especially between fibers of differing (bio)physical properties (Grill and Mortimer, 1996). Simulation of microelectrode stimulation also suggests selectivity between cells and fibers may be possible (Grill and McLntyre, 2001). DBS experiments suggest that changes in the stimulating pulse results in changes in response (Akbar et al., 2016). Boston Scientific has developed research programming software which unlocks additional stimulator capability, without the need to alter device firmware. In particular, the software can control the polarity, amplitude, and pulse-width of additional active stimulation pulses, adding a pre- and post-pulse to the existing stimulation pulse. These pulses can be distributed across the lead electrodes (e.g., a pre-pulse to E1, stimulation to E1 and E2, and a post pulse to E2). Boston Scientific stimulators support combined pulse configurations of up to 12 active phases, enabling clinical testing of pulse shapes previously explored in computational models (Foutz and McIntyre, 2010).

The INSHAPE DBS project, led by KU Leuven, has tested various non-commercial pulses in PD and ET patients in a randomized double-blind crossover design using the above system. For this study, a sensing component was added by including EPs recorded with EEG, and further testing will be performed in an expanded cohort in the chronic condition. The project has investigated 12 different stimulation pulses, combining cathodic, anodic, and biphasic pulses with additional hyperpolarizing pre-pulses of a low, medium, and high amplitude with pulse widths of 120, 240, and 360 μs. Therapeutic window was measured between the minimum current to observe a therapeutic response and the maximum current before observing any side effect. From preliminary data in 4 patients, these investigators found that the different combinations of stimulation pulses had distinct effects in the therapeutic window and that this effect was patient-specific. Additional research will be required to refine this technique toward improving clinical outcomes.

## Neuroethical, Legal, and Socio-Cultural Issues of DBS

As this report, and proceedings from prior Think Tanks illustrate, DBS, like many domains of current and emerging neurotechnology, is advancing, in large part, because of increasingly sophisticated engineering, and expanding knowledge as well as an enhanced understanding of neural systems (Gunduz et al., 2015; Deeb et al., 2016; Rossi et al., 2016; Ramirez-Zamora et al., 2018, 2019). These advances are fostering growing consideration of DBS for studying and treating a broadening scope of neuropsychiatric disorders. Moreover, DBS research and clinical use are becoming ever more international, as several countries are dedicating considerable financial resources to large-scale neuroscientific and neurotechnological initiatives. The field is collaborative and competitive; and both collaboration and competition can evoke asymmetries in technological capability, focus and scope of research, and provision and access to interventions (Martin et al., 2016; Becker et al., 2017; Giordano, 2017). These asymmetries can—and likely will—occur both within nations (e.g., as reflective of differing economics, insurance coverage, etc.,), and between nations (i.e., in light of distinct cultures’ economies, norms, and values, philosophies, ethics, and laws).

Thus, while some ethical issues can be similar or identical (e.g., risks of neurosurgery; inherent uncertainties of new technology), others may not (Martin et al., 2016; Becker et al., 2017). As we have noted, even fundamental ethical concepts (e.g., meanings, value, and questions about autonomy, as affected by DBS such as those described below) can be viewed and regarded through differing cultural lenses (Giordano, 2016). Laudably, several intra- and multi-national groups have committed resources to address the ethical-legal and social issues generated by DBS research and use (for example, the efforts of the United States’ National Institutes of Health; and Asociación Mexicana de Neuroética, as reported in this article, as well as numerous others).

This is vital, given that the internationality of brain science enterprises would require any authentic neuroethics to be insightful, relevant, and responsive to issues arising in and from the development and applications of DBS—and other neurotechnologies—on the global stage (Rossi et al., 2014; Ramirez-Zamora et al., 2018). To effectively approach these challenges—and opportunities—it will be important to establish some international forum for the iterative exchange of ideas (of currently committed programs, and newly emerging projects in neuroethics), that remains apace with worldwide developments, capabilities, and limitations of neuro-engineering and the social sphere.

### The IEEE Brain Initiative Neuroethics Program

Toward such ends, the Institute of Electrical and Electronics Engineers’ (IEEE) Brain Initiative Neuroethics Subcommittee is engaged in a multi-year effort to identify current international trends in neuro-engineering; define uses in various contexts and practices; describe ethical-legal and social implications, issues, questions and problems; and develop guidelines for their responsible address. Bringing together subject matter experts in engineering, anthropology, philosophy, ethics and law (see www.braininitiative.org/alliance/ieee-brain), the project is creating an open-access, web-based (and print) platform that enables interactive discourse and ongoing updates as pertinent to developments in the field—and its spheres of application. Importantly, the project aims to develop: (1) consensus and dialectic among share- and stake-holders in the field; and (2) public visibility and awareness of the process. The project, which began in summer 2018 is aiming toward completion by mid/end-2021 (for more detailed information, see www.braininitiative.org/alliance/ieee-brain).

To be sure, the use of DBS, like any therapeutic approach, is aimed at providing maximum benefit for the good of those patients in need. As DBS gains in both technologic sophistication and relative popularity, we must be cognizant that the use of these technologies and techniques uphold patients’ values and goals. These concerns call special attention to evaluating DBS and its outcomes, incorporating neuroethical assessment and guidance into research, and understanding the use of DBS in and across international contexts of use.

### Control, Personality, and Neuroethical Issues in the Use of DBS for PD

Gisquet (2008) have claimed that DBS is a disruptive experience for some patients due to the associated loss of control of the illness and of one’s life, and the possibility of undesired personality changes incurred by the use of this technology. These concerns generated considerable conceptual neuroethical discourse, however, most publications addressing this topic were not based on empirical data (Frederic Gilbert and Ineichen, 2018). Thus, efforts are underway to employ empirical methods to re-examine assertions that DBS results in diminished control and undesired personality changes. Initial studies focused on DBS for PD systematically solicited patients’ major symptom-reduction and functional expectations and goals for DBS surgery. Changes in symptoms and patients’ perception(s) of control were prospectively assessed at baseline, 3 months post-op, and 6 months post-op. It was found that overall, DBS significantly improved patients’ symptoms and personal goals and that these outcomes were highly meaningful and valuable to them.

Interestingly, despite conversations with multiple team members regarding expectations, symptoms that typically do not respond well to DBS (i.e., non-motor symptoms) were among those most cited as symptom-relevant goals (Kubu et al., 2017). Patients’ underlying motivations to pursue DBS were primarily related to larger life goals including relationships, avocational pursuits, and work. Additionally, patients’ sense of global control of their life significantly increased after DBS, whereas their desire to control their device decreased as patients developed increasing trust in the treatment team and their expertise. Importantly, these initial studies revealed that existing clinical measures do not fully capture patients’ goals and motivations. This is a critical issue for assessing patient-centered care, the effectiveness of DBS, and the clinical team’s performance (Kubu and Ford, 2012; Kubu et al., 2017, 2018). Furthermore, patients’ primary treatment goals changed after DBS, calling into question the idea of how these expectations may change over time, and what this infers for consent.

Current studies are exploring patients’ and family members’ perspectives and experiences of the preservation of their most valued personality characteristics at different stages of PD and over the course of DBS, using a cohort of patients with PD diagnosed less than 1 year prior, diagnosed within 5–7 years prior, and patients approved for DBS. Preliminary results reveal that standard personality measures do not comprehensively assess what matters most to patients. Furthermore, patients retrospectively reported an average decline in valued personality characteristics over the course of PD across all three cohorts. However, patients who are candidates for DBS were significantly more likely to anticipate future gains in valued personality characteristics (closer to their premorbid level), whereas patients who are not candidates for DBS anticipated continued losses of valued personality characteristics over time. Finally, DBS was associated with personality ratings closer to reported historical scores and an increase in global control. In sum, these data refute some of the claims that DBS causes undesired personality changes, provides empirical evidence of what clinical goals and outcomes are most important to patients and families, and these findings highlight the need to develop clinical measures that are more patient-centered and more accurately address and reflect individual patients’ values.

### Updates on the NIH BRAIN Initiative in Neuroethical Issues

As developments in technology and neuroscience introduce novel ethical challenges in research and clinical care, a more contemporary definition of the foci and functions of neuroethics—as a field and set of practices—is needed. Advances in neurotechnology research, development and use make it important to consider emerging questions and implications that such progress foster for research participants, family members, researchers, and the community-at-large. To address these questions and develop a roadmap for future inquiry in neuroethics, the National Institutes of Health (NIH) established an advisory committee under auspices of the Brain Research through Advancing Innovative Neurotechnologies (BRAIN) Initiative. In recognizing the need and value of supporting neuroethical inquiry and address, the NIH has begun to fund research and training projects through a variety of funding mechanisms (e.g., R01s, F32s), and administrative supplements to embed ethicists into BRAIN Initiative-supported research.

As we use novel neurotechnology in clinical trials, several guidelines have been developed, with safety being the most important factor to consider. In this regard, researchers should ensure informed consent processes are attentive towards psychosocial risks (i.e., changes in self-identity, effects of research interventions on interpersonal relationships, and potential shifts in patient values), and should include detailed protocols that address end-of-trial and post-trial responsibilities relevant to physical and psychological risks that may be incurred at post-study follow-up. Additionally, researchers should include a clinician on study teams to enable a better understanding of the clinical implications of research undertaken.

Moreover, it will be important to foster public education to create dialogs to communicate results of research in ways that be broadly understood. Crucial to this pursuit is an awareness of media influence on public views. To maximize the relevance and generalizability of DBS research, it will be even more important to include the public in neuroethical discourse. Finally, but certainly not least, interdisciplinary collaborations should be developed to more ably integrate neuroethical assessment and guidance to ongoing research projects. We believe that each and all of these steps will encourage attentiveness to neuroethical dimensions of DBS research while supporting training opportunities for the next generation of neuroethics, researchers and clinicians.

### Neuroethics in Global Context: The Use of DBS in Mexico

Research and clinical use of DBS are expanding beyond the developed world. This has prompted consideration if and to what extent DBS is cross-culturally valid, pertinent and valuable. As an exemplar, in Mexico, there are particular neuroethical, legal and socio-cultural issues (NELSCI) that might shape perceptions and evaluations of scientific and technological tools and techniques (Karen Herrera-Ferrá et al., 2019). These factors prompt consideration of proactive inclusiveness of diverse ethnocultural contexts and factors (e.g., needs, values, philosophies, beliefs and traditions) within and across countries, in order to better understand various views (i.e., culturally-framed cognition), specific local NELSCI, and attitudes that could direct the use -or non-use- of advanced neurotechnology, such as DBS. Comprehensive cultural competency could—and should—be developed and fortified to provide complementary reflections to enable more meaningful discourse, this will be important to identify and increase clinical goals and benefits, reduce burdens and harm(s), and in these ways, improve global efforts to promote and sustain ethically sound translational—and sensitively *transnational—*use of DBS.

### Summary and Conclusions

The Seventh Annual DBS Think Tank addressed in the most current: (1) commercially available technologies; (2) use of advanced technologies to improve clinical outcomes; (3) research in neuromodulatory approaches to treating neuropsychiatric disorders; (4) use and utility of complex neurophysiological signals for advancing delivery of neurostimulation; and (5) ethical issues arising in and from research and use of DBS. Every year, the attendees of the DBS Think Tank are asked to answer a questionnaire in which they position different neurotechnologies on the Hype Cycle curve (Supplementary Figure S6). Sixty participants responded, the vast majority working at academic institutions and universities. The weighted-mean experience in the field of neurotechnology of the participants is 10 years. In the last year, DBS for Parkinson’s disease and essential tremors remain at the slope of enlightenment. Similarly, vagus nerve stimulator (VNS) uses in obesity, rheumatoid arthritis, heart failure, and stroke are at the technology trigger. Interestingly, low-intensity focused ultrasound moved from technology trigger to peak of inflated expectations, which corresponds with its expanding applications. On the other hand, cochlear implants dropped from the slope of enlightenment to the trough of disillusionment.

These advances serve as both “markers of progress” and challenges and opportunities for ongoing address, engagement, and deliberation as we move to improve the functional capabilities and translational value of DBS. It is in this light that these proceedings are presented to inform the field and initiate ongoing discourse. As consistent with the intent, and spirit of this, and prior DBS Think Tanks, the overarching goal is to continue to develop multidisciplinary collaborations to rapidly advance the field and ultimately improve patient outcomes. Our ongoing work remains dedicated to these efforts.

## Data Availability Statement

The datasets generated for this study will not be made publicly available. Individual investigators will provide datasets at time of publication of specific research.

## Ethics Statement

The studies involving human participants were reviewed and approved by Individual academic institutions. The patients/participants provided their written informed consent to participate in this study.

## Author Contributions

AR-Z, JG, AG, JA, JC, SC, RE, JG, SL, BP, JW, PD, BP, SC, WG, HW, SL, RG, GT, WT, NS, AL, TF, AF, SS, GS, TS, JM, AB, PR, CM, LS, NS, CK, LS, KH-F, SG, BC, GS, CH, LA, WD, KF, and MO fulfilled the authorship criteria by substantial contributions to the conception of the work, providing data for the work, revisiting it critically for important intellectual content, approving the final version, and agreeing to be accountable for all aspects of the work in ensuring that questions related to the accuracy or integrity of any part of the work are appropriately investigated and resolved.

## Conflict of Interest

AR-Z acknowledges support from the Parkinson’s Foundation and has received consulting honoraria from Medtronic, Boston Scientific, Bracket, Stealth, CNS raters, Rho Inc in the past 24 months. AG receives device donations from Medtronic, PLC under collaborative agreements. JGo worked for Medtronic in Colombia as a Pain Sales Representative in 2018 (only for 6 months, Feb-Jul 2018). HW reports no relevant conflicts of interest for this work. Dr. HW is employed by the University of Alabama at Birmingham, and he serves as a clinical scientific consultant for Medtronic and Boston Scientific. WT and NS are named inventors on patents relating to ERNA, which are assigned to DBS Technologies Pty Ltd. WT and NS hold shares and/or options in DBS Technologies Pty Ltd. WT has previously received honoraria from Medtronic and Boston Scientific. AL has served as consultant for Medtronic, Abbott, Boston Scientific and Functional Neuromodulation. TF has received grant funding from Cure Parkinson’s trust, Michael J. Fox Foundation, John Black Charitable Foundation, Defeat MSA, Van Andel Institute, Innovate UK. He has received honoraria for speaking at meetings sponsored by Bial, Profile Pharma and Boston Scientific, and has served on Advisory Boards for Living Cell Technologies, Voyager Therapeutics. None of these institutions had any role in the current manuscript. SS reports no conflict of interest relevant for this work, but he has consulting agreements with Abbott, Zimmer, and Koh Young. RG reports that senior author Dr. Philip Star receives research support from Medtronic Inc. (devices provided at no cost), and funds for fellowship training. US patent #9,295,838 is related in part to this work. LA has worked as an educational and advisory board consultant and has received honoraria from Medtronic and Boston Scientific. SG is an employee and stockholder of Medtronic PLC, and holds patents assigned to Medtronic PLC in the field of neuromodulation. BC is an employee of Abbott. GS is an employee of Boston Scientific Neuromodulation”. MO serves as consultant for the National Parkinson’s Foundation, and has received research grants from the National Institutes of Health, National Parkinson’s Foundation, Michael J. Fox Foundation, Parkinson Alliance, Smallwood Foundation, Bachmann-Strauss Foundation, Tourette Syndrome Association, and UF Foundation. MO has previously received honoraria, but in the past > 60 months has received no support from industry. MO has received royalties for publications with Demos, Manson, Amazon, Smashwords, Books4Patients, and Cambridge (movement disorders books). MO is an associate editor for New England Journal of Medicine Journal Watch Neurology. MO has participated in CME and educational activities on movement disorders (in the last 36 months) sponsored by PeerView, Prime, Quantia, Henry Stewart, and the Vanderbilt University. The institution and not MO receives grants from Medtronic, Abbvie, and ANS/St. Jude, and the principal investigator has no financial interest in these grants. MO has participated as a site principal investigator and/or co-investigator for several NIH foundation, and industry sponsored trials over the years but has not received honoraria.

The remaining authors declare that the research was conducted in the absence of any commercial or financial relationships that could be construed as a potential conflict of interest.

## Footnotes

^1^https://case.edu/hololens/

## Supplementary Material

FIGURE S1 Adaptive Deep Brain Stimulation (aDBS) relies on identifying unique neurophysiological biomarkers using local field potentials (LFPs) at the cortical (using Ecog) or subcortical (through the DBS lead) level to trigger and deliver stimulation using an advanced sensing device (for additional details please refer to main text).

FIGURE S2 Example of a neuronal closed loop-DBS trial: upper panel demonstrates fluctuation of subthalamic nucleus (STN) beta power within, above and below the dual thresholds; lower panel demonstrates the DBS voltage response. The insert highlights the decision events over a 1 s period, whether DBS voltage increased (red triangles), stayed the same (no symbols), or decreased (blue triangles). From Velisar et al. (2019) with permission.

FIGURE S3 Real-time collection of local field potential to enable high accuracy differentiation of clinical states for adaptive neuromodulation (i.e., home environments).

FIGURE S4 DBS evoked resonant neural activity (ERNA). **(A)** Applying stimulation in the vicinity of the STN elicits evoked responses with decaying oscillation morphology. Columns show the ERNA recorded at each electrode when stimulation was applied to different electrodes (indicated by crossed axes) in a person with PD. A 3D reconstruction illustrating electrode positions (green: STN, blue: substantia nigra). **(B)** Normalized ERNA amplitude varies with electrode position with respect to (w.r.t) the STN in people with PD (20 hemispheres tested; box: 25th–75th percentiles; line: median; whiskers: range). ****p* ≤ 0.001, ***p* < 0.01, **p* < 0.05. **(C)** Mean Unified Parkinson’s Disease Rating Scale (UPDRS) improvement from stimulation after ranking electrodes within each hemisphere according to ERNA amplitude (rank 1: largest ERNA; bars: standard error). Results from 10 PD patients tested post-surgery (20 hemispheres). **(D)** ERNA recorded in a person with PD at electrode implantation (blue) and under general anesthesia 560 days postop (red). **(E)** ERNA frequency decreases with increasing DBS amplitude (19 hemispheres tested). Red bars: *p* ≤ 0.001; yellow bars: *p* < 0.05. **(F)** ERNA frequency correlates with relative beta band (13–30 Hz) amplitude across the stimulation levels shown in (**E**; *ρ* = 0.58, *p* < 0.001). Colors represent different hemispheres tested. Panels **(A–D)** from Sinclair et al. (2018) with permission. Panels **(E,F)** from Sinclair et al. (2019) with permission.

FIGURE S5 Illustration of Pathway Generation Process including: **(A)** histological data from the Morel atlas, **(B)** histological data fitted to the CIT 168 brain atlas, **(C)** preliminary pathway trajectories were generated using information from the MRI, histology, and previous literature, **(D)** mean trajectories were generated for each pathway, **(E)** preliminary streamline bundles were generated, **(F)** using the HoloLens system, these pathways were visualized, discussed, and manually edited *via* holographic interactions with the neuroanatomists and **(G)** finalized pathways. From Petersen et al. (2019) with permission.

FIGURE S6 Hype Cycle schematic representation. This Figure represents the position of certain DBS-associated technologies at different stages of development on the Hype Cycle curve (Fenn, 1999).
