# Supplementary figures and images for: Proceedings of the Seventh Annual Deep Brain Stimulation Think Tank: Advances in Neurophysiology, Adaptive DBS, Virtual Reality, Neuroethics and Technology
Source: Front Hum Neurosci. 2020 Mar 27;14:54. doi: 10.3389/fnhum.2020.00054 (PMC7134196; doi:10.3389/fnhum.2020.00054)

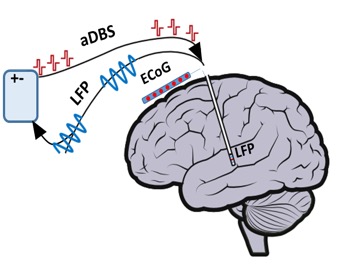

Supplement: FIGURE S1 — Adaptive Deep Brain Stimulation (aDBS) relies on identifying unique neurophysiological biomarkers using local field potentials (LFPs) at the cortical (using Ecog) or subcortical (through the DBS lead) level to trigger and deliver stimulation using an advanced sensing device (for additional details please refer to main text). [file Image_1.jpeg]

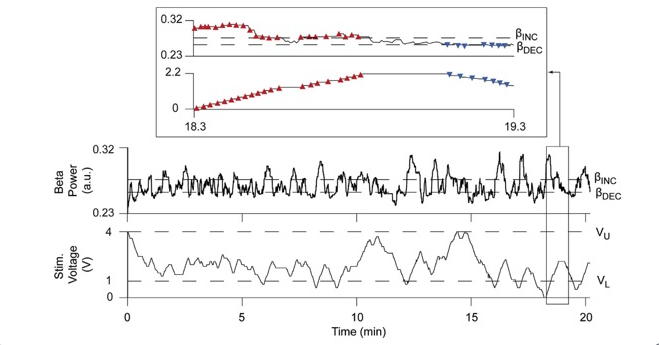

Supplement: FIGURE S2 — Example of a neuronal closed loop-DBS trial: upper panel demonstrates fluctuation of subthalamic nucleus (STN) beta power within, above and below the dual thresholds; lower panel demonstrates the DBS voltage response. The insert highlights the decision events over a 1 s period, whether DBS voltage increased (red triangles), stayed the same (no symbols), or decreased (blue triangles). From Velisar et al. (2019) with permission. [file Image_2.tiff]

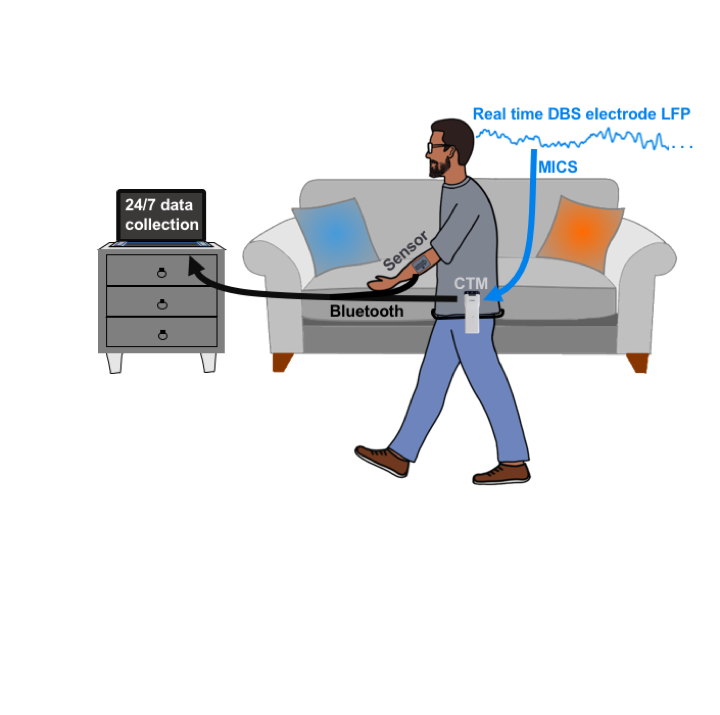

Supplement: FIGURE S3 — Real-time collection of local field potential to enable high accuracy differentiation of clinical states for adaptive neuromodulation (i.e., home environments). [file Image_3.tiff]

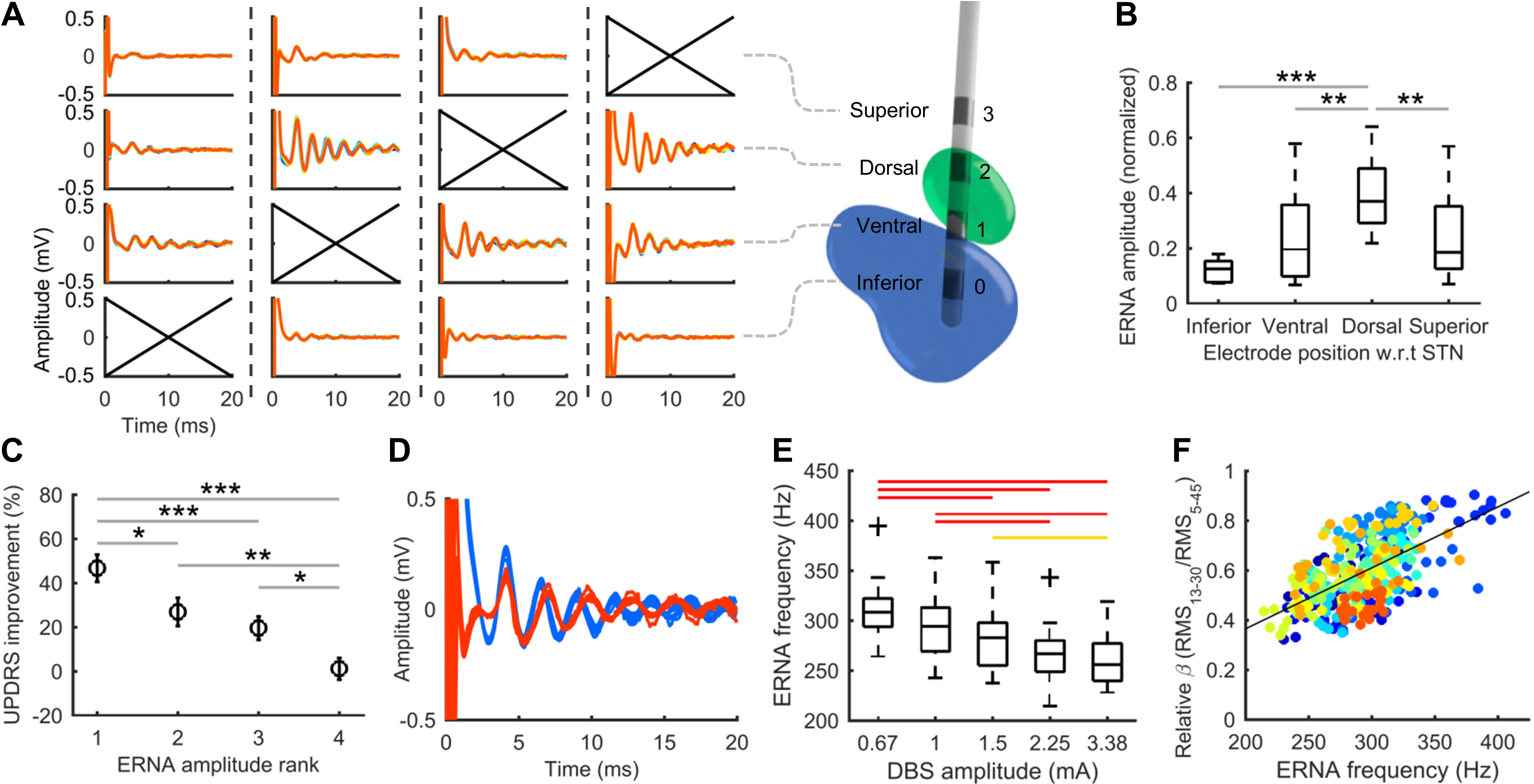

Supplement: FIGURE S4 — DBS evoked resonant neural activity (ERNA). (A) Applying stimulation in the vicinity of the STN elicits evoked responses with decaying oscillation morphology. Columns show the ERNA recorded at each electrode when stimulation was applied to different electrodes (indicated by crossed axes) in a person with PD. A 3D reconstruction illustrating electrode positions (green: STN, blue: substantia nigra). (B) Normalized ERNA amplitude varies with electrode position with respect to (w.r.t) the STN in people with PD (20 hemispheres tested; box: 25th–75th percentiles; line: median; whiskers: range). ***p ≤ 0.001, **p < 0.01, *p < 0.05. (C) Mean Unified Parkinson’s Disease Rating Scale (UPDRS) improvement from stimulation after ranking electrodes within each hemisphere according to ERNA amplitude (rank 1: largest ERNA; bars: standard error). Results from 10 PD patients tested post-surgery (20 hemispheres). (D) ERNA recorded in a person with PD at electrode implantation (blue) and under general anesthesia 560 days postop (red). (E) ERNA frequency decreases with increasing DBS amplitude (19 hemispheres tested). Red bars: p ≤ 0.001; yellow bars: p < 0.05. (F) ERNA frequency correlates with relative beta band (13–30 Hz) amplitude across the stimulation levels shown in (E; ρ = 0.58, p < 0.001). Colors represent different hemispheres tested. Panels (A–D) from Sinclair et al. (2018) with permission. Panels (E,F) from Sinclair et al. (2019) with permission. [file Image_4.tif]

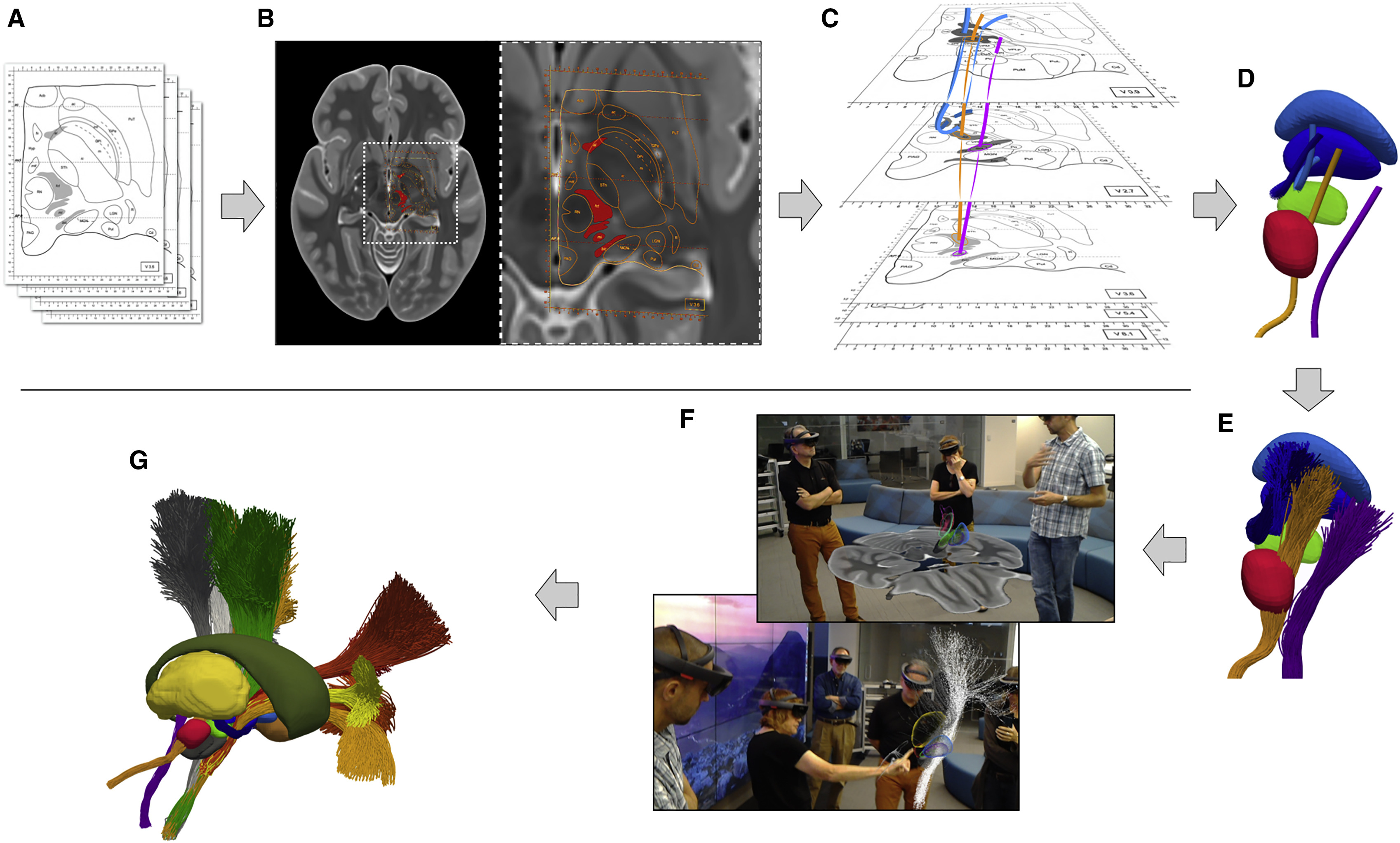

Supplement: FIGURE S5 — Illustration of Pathway Generation Process including: (A) histological data from the Morel atlas, (B) histological data fitted to the CIT 168 brain atlas, (C) preliminary pathway trajectories were generated using information from the MRI, histology, and previous literature, (D) mean trajectories were generated for each pathway, (E) preliminary streamline bundles were generated, (F) using the HoloLens system, these pathways were visualized, discussed, and manually edited via holographic interactions with the neuroanatomists and (G) finalized pathways. From Petersen et al. (2019) with permission. [file Image_5.jpeg]

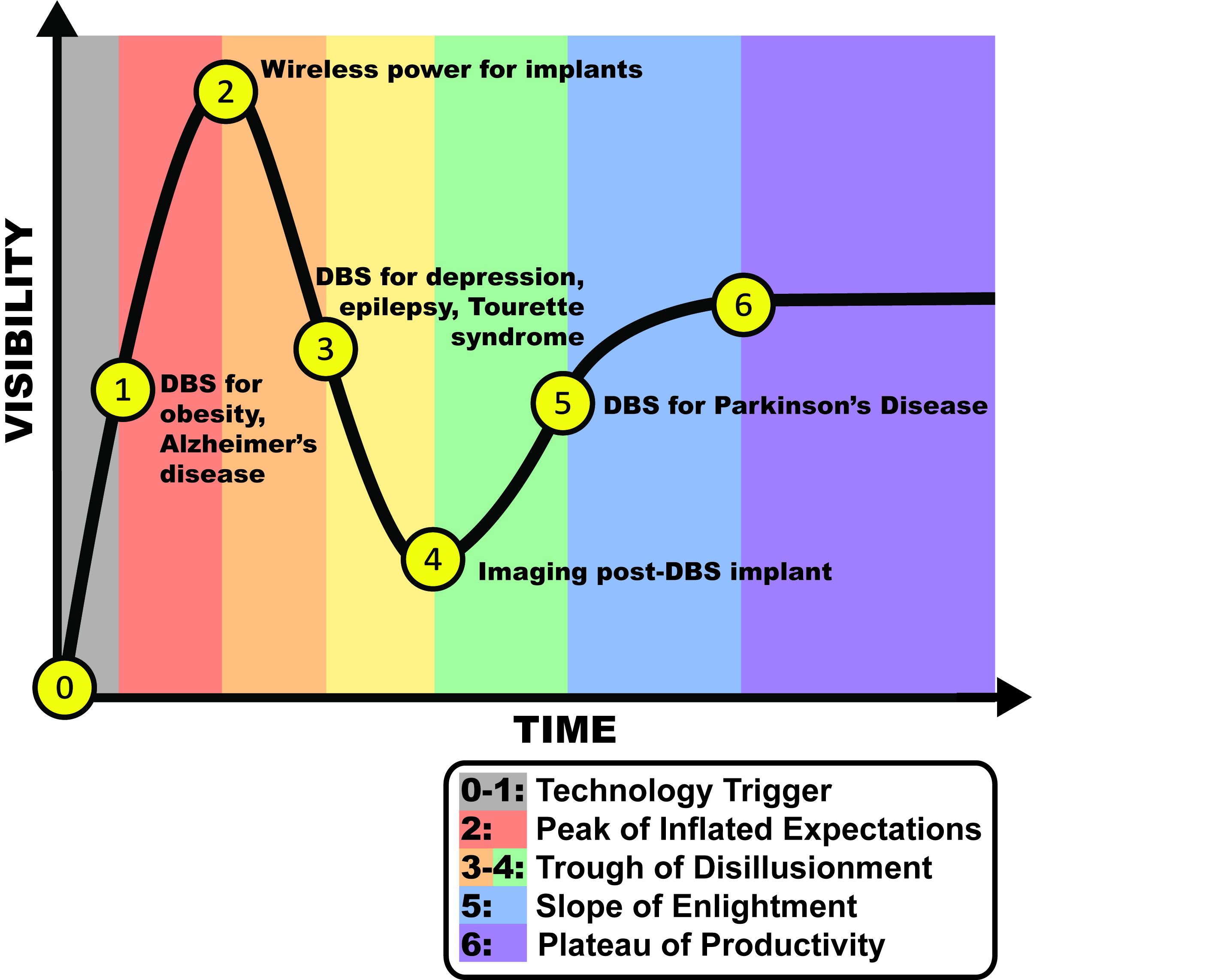

Supplement: FIGURE S6 — Hype Cycle schematic representation. This Figure represents the position of certain DBS-associated technologies at different stages of development on the Hype Cycle curve (Fenn, 1999). [file Image_6.tif]
